# Sexual function in women with androgen excess disorders: classic forms of congenital adrenal hyperplasia and polycystic ovary syndrome

**DOI:** 10.1007/s40618-020-01332-3

**Published:** 2020-06-15

**Authors:** A. Kępczyńska-Nyk, A. Kuryłowicz, A. Nowak, T. Bednarczuk, U. Ambroziak

**Affiliations:** 1 Department of Internal Medicine and Endocrinology, Warsaw Medical University, 1a Banacha street, 02-097 Warsaw, Poland; 2 Department of Human Epigenetics, Mossakowski Medical Research Centre, Polish Academy of Sciences, 5 Pawińskiego street, 02-106 Warsaw, Poland

**Keywords:** Congenital adrenal hyperplasia (CAH), Polycystic ovary syndrome (PCOS), Androgen excess disorders (AED), Sexual function, Androgens

## Abstract

**Purpose:**

We compared the sexual function in women with classic forms of congenital adrenal hyperplasia (CAH) and polycystic ovary syndrome (PCOS) to find if the cause of androgen excess determines sexual functioning.

**Methods:**

Hundred and four women (21 with CAH, 63 with PCOS and 20 healthy controls) aged 18–40 years were included into the study. All participants completed a questionnaire regarding their sociodemographic background and underwent anthropometric and basic biochemical measurements. Plasma levels of total testosterone, androstenedione, and 17-hydroxyprogesterone were measured with immunoassay. To assess the sexual functions, the Female Sexual Function Index (FSFI) questionnaire was applied.

**Results:**

Apart from the higher physical activity in PCOS patients (*P* = 0.017), we found no significant sociodemographic differences between the studied groups. In clinical assessment, women with CAH had a lower incidence of acne (*P* = 0.006). Their plasma levels of 17OHP (*P* = 0.005) and insulin resistance index (*P* = 0.0248) were higher, while total testosterone (*P* = 0.0495) and glucose (*P* = 0.0061) was lower compared to the PCOS group. Significantly more women with CAH were homosexual (*P* = 0.003) and bisexual (*P* = 0.006). CAH group showed a lower total FSFI score (*P* = 0.0043) and lower scores in three domains: lubrication (*P* = 0.0131), sexual satisfaction (*P* = 0.0006), and dyspareunia (*P* < 0.0001). Higher physical activity was associated in all women with higher total FSFI score (*P* = 0.009) and scores in the domain of desire (*P* = 0.034) and sexual satisfaction (*P* = 0.01), while in CAH women apart from the total score (*P* = 0.03) and sexual satisfaction (*P* = 0.002) also in the domains of orgasm (*P* = 0.005), and pain (*P* = 0.03).

**Conclusions:**

CAH women present more often homosexual and bisexual orientation, while their sexual functions are impaired compared to PCOS patients.

**Electronic supplementary material:**

The online version of this article (10.1007/s40618-020-01332-3) contains supplementary material, which is available to authorized users.

## Introduction

Androgen excess is one of the most common endocrine disorders affecting approximately 7% of reproductive-age women [1, 2]. It negatively influences the quality of life as well as sexual outcomes. The most commonly diagnosed condition associated with hyperandrogenism is polycystic ovary syndrome (PCOS), diagnosed in approximately 70–80% of the patients [3]. Congenital adrenal hyperplasia (CAH), a term applied to a group of autosomally inherited recessive disorders caused by mutations in a single enzyme involved in cortisol synthesis, is much less common. Classic forms of the disease are usually diagnosed in neonates, if diagnosed in adulthood, concerns approximately 0.69% of women with androgen excess disorders (AED) [4]. In 90%, there is a defect in 21-hydroxylase synthesis resulting from mutation of the *CYP21A2* gene. The enzyme deficiency results in a severe deficiency of cortisol and aldosterone, or solely cortisol, as well as an increased level of androgens [5].

There are three main forms of the disease depending on the degree of enzyme deficiency: classic forms—salt-wasting (SW CAH) and simple virilising (SV CAH) and non-classic form (NC CAH) diagnosed in adulthood. Increased level of androgens during early embryonic development leads to various degrees of virilisation of external genitals in females. In severe forms of sexual ambiguity, there might be a difficulty with sex assignment. Girls undergo extensive surgical management to correct clitoris, vulva, and vaginal anatomy. Feminising surgery aims to create female-appearing external genitalia to benefit a normal psychosexual development and functional vagina to allow menstruation and sexual activity. CAH girls of any subtype may develop chronic virilisation, muscle hypertrophy, acne, hirsutism, male pattern alopecia, menstrual abnormalities, as well as side effects of chronic corticosteroid therapy (which is a treatment of choice in CAH). Although girls and adult women with CAH manifest increased gender atypical behavior (which correlates with the severity of *CYP21A2* genotype), most of them develop their gender identity as women [6]. Their sexual orientation is usually heterosexual, but in this group, homosexual and bisexual orientation were observed more frequently than in the general population [7]. Female patients show a vast spectrum of sexual outcomes. They still suffer major limitations in their sexual function and reproductive life. Sexual dysfunction partly depends on vaginal penetration, lack of lubrication, pain, and lack of clitoral sensitivity, but also embarrassment about atypical genitalia and anxieties about sexual performance [8]. Sexual debut in this group tends to occur at older ages, and there are fewer pregnancies and children born [9]. An important issue is the patients’ perception of the disease, which is dependent on family support and social relationships. If a patient has this kind of support, it positively influences the quality of life as well as sexual outcomes [10].

Patients with PCOS show symptoms of androgen excess, menstrual abnormalities, and metabolic syndrome, similar to patients with CAH. PCOS is also associated with impaired sexual functions [11]. Women with this syndrome report worse marital functioning, less satisfaction with their sex life, and rate themselves negatively as sexual partners [12].

Both CAH and PCOS show a similar clinical picture like hyperandrogenism, metabolic syndrome, and menstrual abnormalities, and both can be associated with sexual dysfunction compared to women without AED. However, there is a lack of data describing the sexual function of CAH women in comparison to PCOS women. Therefore, in our study, we decided to compare sexual functions in patients suffering from those two diseases which have a different origin, in reference to healthy women without hyperandrogenism, to establish if pathogenesis of AED has an influence on sexual life in women.

## Materials and methods

### Study population

Eighty-four women (18–40 years old) diagnosed and treated in the Department of Endocrinology of Warsaw Medical University between January 2011 and December 2015 were recruited to the study. In this group, 21 patients were diagnosed with CAH in a prenatal or neonatal period, while in the remaining 63, PCOS was diagnosed based on the Rotterdam criteria [13].

Among CAH patients, 20 were diagnosed with 21-hydroxylase defect: in 17 salt-wasting form of CAH was present (CAH SW) while in three—simple virilisation form of CAH (CAH SV). In one patient, 11-beta-hydroxylase deficiency was diagnosed. Three mothers of CAH patients were treated with dexamethasone during pregnancy; in two of the newborns, genital virilisation was absent, in one genitals were virilised (Prader stage II). All but two CAH patients mentioned above without virilisation had two or more early (first years of life) surgical correction of external genitals, one was operated in 13rd, one in the 26th year of life. All CAH patients were treated with corticosteroids (prednisone 5–10 mg or hydrocortisone 30–40 mg daily). Additionally, SW CAH patients were treated with fludrocortisone (0.5 mg daily).

Patients with a final diagnosis of PCOS were admitted to hospital for evaluation of symptoms potentially related to androgen excess: oligo/amenorrhoea, excessive hair growth, alopecia, and acne. Pelvic and adrenal ultrasound (US) examinations were performed in all patients. In the case of adrenal tumor suspicion on the US, computed tomography of adrenals was also performed. All PCOS patients had hyperandrogenemia and/or clinical signs of it. During the recruitment for the study, none of the PCOS patients received any treatment.

The control group consisted of 20 women aged between 18 and 40 years with regular menstrual cycles and no clinical signs of hyperandrogenism (androgenetic alopecia/hirsutism/acne). Although their adipose tissue content was not calculated, based on their medical history, body mass index (BMI), biochemical parameters, as well as an absence of any components of metabolic syndrome, they were considered to be metabolically healthy.

Exclusion criteria for all studied groups were: (i) lack of patient’s informed consent to participate in the study; (ii) history of mental disorders; (iii) history of chronic diseases; (iv) presence of hypercortisolemia, hyperprolactinemia, and thyroid dysfunction; (v) pregnancy and/or child delivery less than a year ago; (vi) recent history (until 3 months before entering the study) of hormonal contraceptive, hormonal replacement, or anti-androgenic therapy.

All study participants stayed in a relationship and engaged in penetrative sex. None underwent sexual counseling or therapy, but all declared a wish to improve their sexual functioning.

### Biochemical and hormonal measurements

Fasting venous blood samples were drawn from the antecubital vein, in a quiet, temperature-controlled room from 7.30 to 8.30 am to avoid possible circadian fluctuations in the studied parameters. Assessment of total testosterone (TT), sex-hormone-binding globulin (SHBG), albumin, glucose, high-density lipoprotein (HDL), triglycerides (TG), alanine aminotransferase (ALT), and insulin concentrations were performed using ECLIA Cobas diagnostic kits (Roche Diagnostics GmbH, Mannheim, Germany). 17-Hydroxyprogesterone (17OHP) was measured with the ELISA NovaTec test (NovaTec Immunodiagnostica GmbH, Dietzenbach, Germany), androstenedione (A)—with ELISA DRG test (DRG Instruments GmbH, Marburg, Germany) (reference values are presented in Table 1). Free testosterone (FT) was calculated from TT, albumin, and SHBG concentrations using the formula available at https://www.issam.ch/freetesto.htm. Insulin resistance index (HOMA-IR) was calculated according to the formula: [fasting insulin (µIU/ml) × fasting glucose (mg/dl)/18 × 22.5]. HOMA-IR > 2 was diagnostic of insulin resistance.

**Table 1:** Sociodemographic, clinical characteristics, and plasma androgen and metabolic parameters in the studied population

	CAH	PCOS	Controls	*P* value	*P* value	*P* value
				CAH vs. PCOS	CAH vs. Controls	PCOS vs. Controls
Patients, *n*	**21**	**63**	**20**			
Age (years)						
Mean (SD)	29.29 (7.59)	26.56 (5.45)	30.85 (6.73)	0.221	0.264	0.154
BMI (kg/m^2^)						
Mean (SD)	25.92 (6.98)	27.58 (6.98)	24.18 (4.37)	0.303	0.098	0.982
Waist circumference (cm)						
Mean (SD)	88.29 (13.25)	87.52 (15.96)	77.26 (10.57)	0.806	0.018	0.011
% adipose tissue						
Mean (SD)	33.45 (8.7)	36.02 (8.43)	NA	0.356	–	–
Physical activity, *n* (%)	5 (23.80)	23 (36.50)	10 (50.0)	**0.017**	0.079	0.282
Smokers, *n* (%)	5 (23.80)	22 (34.92)	3 (15.0)	0.480	0.476	0.091
Education, *n* (%)						
Primary or vocational	7 (33.33)	10 (15.87)	3 (15.0)	0.060	0.166	0.999
Secondary	3 (14.28)	15 (23.80)	3 (15.0)	0.460	0.999	0.404
University education	9 (42.85)	18 (28.57)	9 (45.0)	0.190	0.999	0.179
Student	2 (9.52)	20 (31.74)	5 (25.0)	**0.020**	0.188	0.566
Occupational activity, *n* (%)	19 (90.47)	62 (98.41)	19 (95.0)	0.753	0.578	0.385
Blue-collar	7 (36.84)	20 (32.25)	3 (15.0)	1.000	0.172	0.145
White-collar	11 (57.89)	36 (58.06)	14 (70.0)	0.743	0.248	0.306
Pink-collar	1 (5.26)	6 (9.67)	2 (10.0)	0.413	0.519	0.999
Marital status, *n* (%)						
Married	6 (28.57)	22 (34.92)	8 (40.0)	0.761	0.441	0.681
Not-married	15 (71.42)	41 (65.07)	12 (60.0)			
Deliveries, *n* (%)	4 (19.04)	10 (15.87)	5 (25.0)	0.735	0.645	0.355
Abortions, *n* (%)	3 (14.28)	6 (9.52)	2 (10.0)	0.626	0.413	1.000
Sexual orientation, *n* (%)						
Heterosexual	12 (57.14)	61 (96.82)	20 (100.0)	**0.0001**	**0.0001**	0.287
Homosexual	5 (23.80)	2 (3.17)	0 (0)	**0.003**	**0.006**	0.287
Bisexual	4 (19.04)	0 (0)	0 (0)	**0.006**	**0.013**	1.000
Hirsutism*, *n* (%)	8 (38.09)	49 (77.77)	0 (0)	0.15	**0.0003**	**< 0.0001**
Acne, *n* (%)	3 (14.28)	36 (57.14)	0 (0)	**0.0006**	**0.033**	**< 0.0001**
Alopecia**, *n* (%)	2 (11.74)	10 (15.87)	0 (0)	0.47	0.084	0.014
Menstrual abnormalities, *n* (%)	7 (41.17)	41 (72.21)	0 (0)	**0.0002**	**0.0007**	**< 0.0001**
Genital surgery, *n* (%)	19 (90.47)	Not concerned	Not concerned			
Total testosterone [*N*: 0.10–1.42 nmol/l]						
Median (25–75 percentile)	0.76 (0.09–3.0)	1.79 (1.24–2.28)	1.06 (0.9–1.35)	**0.0495**	0.981	**< 0.0001**
Free testosterone [%]						
Median (25–75 percentile)	1.36 (1.23–1.55)	1.71 (1.33–1.93)	1.4 (1.08–1.66)	0.07	0.8331	0.4309
Androstenedione [*N*: 0.3–3.30 ng/ml]						
Median (25–75 percentile)	2.1 (0.41–6.8)	3.51 (2.44–4.44)	2.1 (1.52–2.89)	0.0631	0.7404	**0.0006**
17OHP [*N*: 0.2–1.3 ng/ml]						
Median (25–75 percentile)	4.80 (1.41–20)	2.01 (1.58–2.66)	1.19 (0.82–1.69)	**0.005**	**0.0009**	**0.0002**
Glucose [*N*: 60–99 mg/dl]						
Median (25–75 percentile)	82 (76.5–87.5)	87 (82–92)	88 (83–94)	**0.0061**	**0.0074**	0.4980
Insulin [*N*: 2.6–24.9 μIU/ml]						
Median (25–75 percentile)	11.75 (5.46–18.22)	8.4 (5.36–14.16)	5.73 (3.78–7.55)	0.2012	**0.0075**	**0.0206**
HOMA-IR						
Median (25–75 percentile)	3.59 (2.64–5.92)	1.84 (1.14–3.2)	1.21 (0.86–1.68)	**0.0248**	0.0933	**0.0355**
ALT [*N*: 7–56 U/l]						
Median (25–75 percentile)	22 (18.5–31.25)	21 (16–30)	17 (12.25–25.25)	0.4194	0.0742	0.2705
HDL [*N*: > 45 mg/dl]						
Mean (SD)	57.93 (14.48)	56.96 (15.36)	69.76 (17.12)	0.9371	0.0706	**0.0105**
TG [*N*: 50–150 mg/dl]						
Median (25–75 percentile)	87 (59–128)	89 (67.5–143)	71 (47.5–89.5)	0.4991	0.1115	**0.0135**

Significant differences are bolded

*17OHP* 17-hydroxyprogesterone, *ALT* alanine transaminase, *BMI* body mass index, *CAH* congenital adrenal hyperplasia, *HDL* high-density lipoproteins, *HOMA-IR* insulin resistance index [fasting insulin (µIU/ml) × fasting glucose (mg/dl)/18 × 22.5], *N* normal range, *NA* not available, *PCOS* polycystic ovary syndrome, *SD* standard deviation, *TG* triglycerides

*Ferriman–Gallwey score ≥ 8 points, **Ludwig scale ≥ 1 stage

### Anthropometric measurements

Anthropometric measurements (weight, height, and waist circumference), the presence of acne, hirsutism, androgenic alopecia, and virilisation were assessed in all patients. Body composition was estimated by the bioelectrical impedance method with OMRON manual device in all women with AED. Menstrual cycles shorter than 28 and longer than 35 days were considered abnormal [14]. Hirsutism was defined by the Ferriman–Gallwey score ≥ 8 [15], androgenic alopecia by the Ludwig scale. The presence of acne and/or virilisation was recorded but not scored.

### Questionnaires

All study participants were asked to complete a questionnaire assessing their demographic characteristics, education, marital status, children delivery, physical activity, smoking, and medical and sexual histories. Women were considered physically active when they trained 2 h a week (gym, running, riding bike, and swimming). All were asked to fulfill the Female Sexual Function Index (FSFI) questionnaire, a validated test evaluating all phases of the female sexual cycle in the past 4 weeks [16, 17], in the first 2–5 days of the menstrual cycle. The questionnaire consists of 19 items divided into six collective subscales (domains): sexual desire (questions 1–2), sexual arousal (questions 3–6), lubrication (questions 7–10), orgasm (questions 11–13), sexual satisfaction (questions 14–16), and dyspareunia (questions 15–19). Each answer is rated on a scale from 0 to 5 or from 1 to 5, where 0 indicates no sexual activity in the past month. Scores for each of the six domains are calculated by summing up individual domain question scores and multiplying the result by the domain factor (i.e., 0.6 for desire, 0.3 for arousal and lubrication, and 0.4 for orgasm, satisfaction, and pain). The minimum possible score to achieve was 2 and maximum 36 points. A higher score in each domain indicates a better status. A summary score below 26.55 indicates sexual dysfunction [18].

### Statistical analysis

Shapiro–Wilk test was used to estimate the normality of the studied groups for statistical analyses. In data, where normal distribution was observed, results were presented as arithmetic mean (± standard deviation, SD), and comparison between the groups was performed using the Student’s *t* test for independent samples. For remaining data, where abnormal distribution was observed, results were presented as the median (25 and 75 percentile). The nonparametric Mann–Whitney test was used for the calculation of those data. Correlation analysis was performed with the Spearman correlation test.

## Results

### General characteristics of study groups

The detailed characteristic of studied groups is presented in Table 1. The groups were comparable in age, BMI, waist circumference, body composition, and smoking habits. They had a comparable sociodemographic background, including education (however, there were more students in the PCOS group, *P* = 0.02) and occupational activity. There were no differences in marital status, children delivery, and abortion. All study participants stayed in a relationship.

The two groups of women with hyperandrogenism differed in physical activity and frequency of menstrual abnormalities. Clinical features of hyperandrogenism (acne) were present more often in the PCOS group than in the CAH group (57.14% vs. 14.28%, *P* = 0.0006). PCOS women had higher TT (*P* = 0.0495) and glucose (*P* = 0.0061) plasma levels. 17OHP plasma levels were higher in CAH patients (*P* = 0.005), as well as HOMA-IR (*P* = 0.0248). No differences in A, FT, HDL, TG concentrations, and ALT activity were observed between the two groups of patients with AED. PCOS patients, compared to the women without hyperandrogenism, had significantly higher concentrations of TT (*P* < 0.0001), A (*P* = 0.0006), and 17OHP (*P* = 0.0002) and worse metabolic profile characterized by higher fasting insulin (*P* = 0.206), HOMA-IR (*P* = 0.0355), TG (*P* = 0.0135), and lower HDL (*P* = 0.0105) levels. As expected, compared to the healthy controls, patients with CAH had higher concentrations of 17OHP (*P* = 0.0009). Surprisingly their median glucose levels were lower (*P* = 0.0074), but this was achieved by significantly higher fasting insulin concentrations (*P* = 0.0075) than in the women without AED.

There were significant differences in sexual orientations between the studied groups: 5 individuals (23.8%) from the CAH group had a homosexual orientation, and 4 (19.04%) were bisexual (all homosexual and bisexual individuals were diagnosed of SW CAH), while in the PCOS group, there were no bisexual individuals and only 2 patients (3.17%) were homosexual. All members of the control group declared to be heterosexual.

### Assessment of sexual functions by FSFI

Women with CAH had significantly lower scores on the FSFI scale compared to women with PCOS. Sexual dysfunction (total FSFI score no higher than 26.55) was diagnosed in 17 (80.95%) women with CAH and 21 (33.33%) PCOS women (*P* = 0.0001). The total FSFI score was lower in women with CAH (*P* = 0.0043). Women with CAH obtained lower scores in three domains: lubrication (*P* = 0.0131), sexual satisfaction (*P* = 0.0006), and dyspareunia (*P* < 0.0001). Results in the remaining three (desire, arousal, and orgasm) were not statistically significant between the two groups of patients with hyperandrogenism (Table 2).

**Table 2:** Female sexual function assessed by the Female Sexual Function Index (FSFI) questionnaire in the studied groups (results are shown as median [25–75 percentile])

Domain	CAH	PCOS	Controls	*P* value	*P* value	*P* value
				CAH vs PCOS	CAH vs Controls	PCOS vs Controls
FSFI total	22.4 [4.59–25.1]	28.5 [23–31.3]	24.9 [15.3–31.1]	**0.0043**	0.1513	0.3088
FSFI 1–2 (desire)	3 [3.6–4.8]	3 [3.6–4.8]	3.6 [2.4–4.8]	0.6851	0.8245	0.6282
FSFI 3–6 (arousal)	3.6 [0.3–4.8]	4.5 [3.6–5.4]	3.9 [1.3–5.1]	0.0624	0.6886	0.3094
FSFI 7–10 (lubrication)	3.6 [0–5.4]	5.1 [4.2–6]	5.4 [2.1–6]	**0.0131**	0.1242	0.6628
FSFI 11–13 (orgasm)	4.4 [0–5.4]	4.4 [3.2–5.2]	3.8 [1.6–5.2]	0.5651	0.8487	0.4492
FSFI 14–16 (sexual satisfaction)	3.6 [0.8–4.8]	4.8 [4–6]	4.2 [2.9–5.4]	**0.0006**	**0.0495**	**0.0313**
FSFI 17–19 (pain)	1.2 [0–4]	5.6 [3.6–6]	4.8 [4–6]	**< 0.0001**	**0.0021**	0.4041

Significant differences are bolded

*CAH* congenital adrenal hyperplasia, *PCOS* polycystic ovary syndrome

Even though the prevalence of sexual dysfunction did not differ significantly between the patients with PCOS and women without AED, the first group had a higher total FSFI score (*P* = 0.0308), as well as a better score in the domain of sexual satisfaction (*P* = 0.0313). When compared to the healthy controls, patients with CAH had lower scores in domains regarding sexual satisfaction (*P* = 0.0495) and dyspareunia (*P* = 0.0021).

In all studied women, physical activity was associated with better sexual functioning in the domain of desire (median for active—4.5, for non-active—3.6, *P* = 0.034), sexual satisfaction (median for active—5.2, for non-active—4.4, *P* = 0.01), and total FSFI score (median for active 30.15, for non-active 24.4, *P* = 0.009). Especially in CAH women, physical activity was associated with better sexual functioning, too. They had higher scores in total FSFI (median for active—33.1, for non-active—18.7, *P* = 0.03), domain of orgasm (median for active—5.6, for non-active—2.8, *P* = 0.005), sexual satisfaction (median for active—6, for non-active—2.4, *P* = 0.002), and also in domain of pain (median for active—5.2, for non-active—0, *P* = 0.03).

In the CAH group, also homosexual orientation was associated with better sexual functioning in the domain of sexual arousal (median for heterosexual—1.6, for homosexual—4.8, *P* = 0.037). Marital status was connected with better sexual functioning in the domain of dyspareunia in CAH women (median for single—0, for couple—3.8, *P* = 0.049) and in the control group (median for single—4.2, for couple 23.4, *P* = 0.0006).

TT, A, 17OHP levels did not differ significantly (*P* > 0.05) in the subgroups of study participants subdivided according to the presence or absence of sexual dysfunction (total FSFI score < 26.55 or ≥ 26.55, Supplementary Table S1). There were no correlations between sexual function and sociodemographic factors such as education, occupational activity, and smoking. Neither any correlations between anthropometric measurements, other laboratory findings, and sexual functioning were found in both groups.

## Discussion

In this study, we compared sexual functions in patients suffering from diseases which show similar symptoms but have a different origin. The main finding is that despite the common clinical picture, sexual functions in women with CAH are impaired compared to PCOS women.

Our patients were examined in a broad, sociodemographic, anthropometric, hormonal, and metabolic context. Both study groups (women with AED) and the control group consisting of healthy women occurred to be relatively homogenous—they did not differ significantly in the aspect of age, demographic status, education, and anthropometric measurements. The main observed difference was abnormal genitalia at birth and surgical procedures in the early childhood in most of the women with CAH and history of long-lasting glucocorticoid treatment in this group, which may influence some of our outcomes. Women with PCOS had a higher frequency of menstrual abnormalities and glucose plasma levels. In the hormonal assessment, women with CAH had higher 17OHP levels—which, in turn, is a marker of the disease and higher frequency of insulin resistance that can be explained by the chronic treatment with glucocorticosteroids. The patients with PCOS had higher total testosterone concentrations; however, the free testosterone levels were comparable between the two groups.

Unlike PCOS women, CAH patients are exposed to a high intrauterine androgen concentration. It determines the development of masculinised external genitalia and also influences some aspects of their psychosexuality. According to the previous reports also in our group of CAH women, all presented female gender identity. Androgen exposure in fetal life may, however, influence sexual orientation. Most women with CAH are heterosexual, but an increased (from 25 up to 50%) prevalence of homo and bisexual orientation has also been reported and positively correlated with the severity of *CYP21A2* mutation [6, 7]. In our CAH group, where data of severity of mutation in the *CYP21A2* gene are incomplete, 43% (all diagnosed with SW CAH, among them there were no patients whose mothers were treated with dexamethasone during pregnancy) declared homo- or bisexual orientation, which is a relatively large number. In the PCOS group, 3.17% were homosexual, and it is less than in the CAH group but more prevalent than in the general population of healthy Polish women, where 1% is considered to be homosexual [19]. Self-identified lesbians are reported to have a significantly higher prevalence of PCO and PCOS compared with heterosexual women [20]. However, an association of sexual orientation with elevated levels of androgen concentrations in PCOS women is questionable, as generally hyperandrogenaemia is observed in the pubertal and postpubertal periods.

Based on the FSFI questionnaire, we found that sexual functions in women with CAH are impaired in comparison with PCOS women. There is a lack of data comparing the sexual functions of these two groups so far, but separately, both CAH and PCOS patients, compared to healthy controls, were reported to have worse scores in FSFI domains of arousal, lubrication, sexual satisfaction, and pain [21–23]. In our study, this finding was right in the case of CAH patients only, who, compared to the healthy controls, had lower scores in domains of sexual satisfaction and pain, while to the women with PCOS—also in the domain of lubrication. Vaginal penetration difficulties, pain, lack of lubrication associated with genital abnormalities, and their surgical correction could be the explanation [8, 24]. However, surgical techniques have changed over the years, and methods of feminising genitoplasty have gradually improved. Therefore, there are data, where sexual functions (also measured by FSFI) in CAH patients are not significantly different from their healthy counterparts [25]. There are also other than genital abnormalities possible reasons for lower quality of sexual life in CAH patients. In our group, mothers of three individuals were treated with dexamethasone during pregnancy, two of them had normal female genitalia, and one had only clitoromegaly (Prader stage II). Their sexual functioning was not better than remained CAH patients, and all were diagnosed with sexual dysfunction (total FSFI < 26.55). There are reports that CAH patients with classical and non-classical forms of the disease, irrespective of history of genital surgery, report problems with intercourse [21, 22]. A comparison of sexual experiences between young adult women with CAH to young adult women with diabetes mellitus concluded that psychological factors noted in women with CAH impacted the sexual outcome in addition to the anatomic concerns [26]. Later sexual debut and fewer pregnancies in CAH girls may be a result of problems with acceptance of their genital appearance and anxieties about sexual performance [8]. We should also consider psychological factors coming from the stigmatization of the disease, more than PCOS patients’ experience. Data about the perception of the disease by our CAH patients, social and family support, and psychological care are not known. Moreover, a neuropsychological function can be influenced by long-term glucocorticoid administration, which can lead to mood disorders, most often depressive symptoms, and, perhaps also, indirectly may disturb sexual functioning [27]. All study participants declared no mood disorders, but it was not estimated by objective examination.

However, hyperandrogenic women diagnosed with PCOS may also deal with problems with feminine identity and self-esteem as they believe that their outer appearance makes it difficult to form social contacts and affects their sexual life. Decreased quality of life, psychological distress, and mood disturbances, including symptoms of depression and decreased sexual satisfaction, have also been reported in these patients [28, 29]. PCOS women find themselves less sexually attractive, partly due to excessive body hair that might impair their sexual life [28–31]. In our group, where the acne was more prevalent in PCOS patients, we did not observe a connection between the presence of symptoms of hyperandrogenism and sexual functioning.

Surprisingly, in our study group, patients with PCOS had significantly better total FSFI punctation and higher scores in the sexual satisfaction domain compared to healthy controls. There is still a discussion in progress about the influence of androgens on sexual functions. Desire and orgasm are considered to correlate positively with testosterone levels [32], so high testosterone levels in PCOS women might reverse the adverse effect of the disease on those domains. In turn, in our study, CAH patients had a lower level of total testosterone than PCOS that might be due to the long-term treatment with glucocorticosteroids. However, we did not find any differences in the domains of desire and orgasm between these two groups. The possible explanation is that the studied groups did not differ in the concentration of free testosterone, which is an active form of the hormone. Moreover, some authors noted that not biological levels of androgens, but clinical signs are predictive of sexual desire [33, 34]. In our group, symptoms of hyperandrogenism, suggesting sensitivity to androgens like acne, and hirsutism were more prominent in PCOS patients, but did not influence their sexual functions.

Our study revealed that physical activity was positively correlated in all women with total FSFI scores and scores in the domains of desire and sexual satisfaction while in the CAH group in the domains of orgasm, sexual satisfaction, and dyspareunia. In general, population exercising, among other factors, consistently has a significant, protective effect on sexual functioning across all domains [35, 36]. Similar findings were noticed in the PCOS population, where physical resistance training significantly enhanced the total score and the desire, arousal, and lubrication domains of the FSFI. It reduced pain, total depression, and anxiety scores in PCOS and also control group of healthy individuals [37]. Exercise programs in women suffering from sexual dysfunction, with AED, including CAH patients, might be one of the treatment options introduced to them.

Interestingly, our study revealed that homosexuality in CAH women was associated with better sexual functioning in the domain of sexual arousal. In Poland, where religious aspects are very important, being homosexual is a kind of distress, and a sexual orientation which is not accepted by the Catholic Church may not positively influence sexual functioning. Some data suggest that dysfunctional sexual beliefs may play a role as vulnerability factors for sexual dysfunction, and lesbians are scoring higher on sexual desire as a sin, age-related beliefs, and affection primacy while lower—on beliefs related to motherhood primacy [38]. However, other studies indicate a relatively similar pattern in the prevalence of sexual problems in lesbians and heterosexual women, and both groups report pain as the most frequent sexual problem. They also emphasize the role of associated levels of distress to self-perceived sexual problems in women, regardless of sexual orientation [39].

Our study has several limitations that should be mentioned. First, even though FSFI was designed to be applicable in women regardless of their sexual orientation, it has not been specifically validated in bi- and homosexual women, which constituted 10.6% of the whole studied group and 42.8% of the CAH patients [40]. Next, according to the Diagnostic and Statistical Manual of Mental Disorders 5 (DSM-5) and International Statistical Classification of Diseases and Related Health Problems (ICD) 10 and 11, one cannot diagnose sexual dysfunction with no accompanying distress [41, 42], while the participants of our study were not, before completing the FSFI questionnaire, diagnosed for the occurrence of distress. However, given the fact that all of them were in a relationship, and all declared a wish to improve their sexual functioning, it is likely that the low scores in FSFI can be attributed to sexual dysfunction. Finally, due to the limited number of study participants, our findings should be treated as preliminary and verified in subsequent studies on larger groups.

In conclusion, analysis of patients with AED of different origins in a broad, sociodemographic, anthropometric, hormonal, and metabolic context revealed that sexual functions in women with CAH are impaired in comparison with PCOS women. Sexual functioning in CAH women depended on the presence of genital abnormalities and surgical procedures but also psychological factors. In studied groups, physical activity improved sexual functioning regardless of AED origin. Additionally, we found that significantly more women with CAH compared to the PCOS patients were homosexual.

## Electronic supplementary material

Below is the link to the electronic supplementary material.

Supplementary file1 (DOCX 15 kb)
