# Supplementary material for: Sexual function in women with androgen excess disorders: classic forms of congenital adrenal hyperplasia and polycystic ovary syndrome
Source: J Endocrinol Invest. 2020 Jun 15;44(3):505–13. doi: 10.1007/s40618-020-01332-3 (PMC7878262; doi:10.1007/s40618-020-01332-3)
Supplement: Supplementary file 1 — Supplementary file1 (DOCX 15 kb) [file 40618_2020_1332_MOESM1_ESM.docx]

**Supplementary Table S1.** Total testosterone, androstendione and 17OHprogesterone levels in the subgroups of study participants subdivided according to the presence or absence of sexual dysfunction (total Female Sexual Function Index (FSFI) < 26.55 or ≥ 26.55). Results shown as median [25-75 percentile]. P value for all comparisons > 0.05.

|  | **Total testosterone**  **[N: 0.10 – 1.42 nmol/l]** | **Androstendione**  **[N: 0.3 – 3.30 ng/ml]** | **17OHprogesterone**  **[N: 0.2 – 1.3 ng/ml]** |
| --- | --- | --- | --- |
| **PCOS FSFI < 26.55** | 1.84 [1.185-2.36] | 3.6 [3-4.44] | 2.04 [1.61-2.93] |
| **PCOS FSFI ≥ 26.55** | 1.6 [1.2-2.17] | 3.305 [2.338-4.413] | 1.87 [1.493-2.588] |
| **CAH FSFI < 26.55** | 0.73 [0.09-1.9] | 2.1 [0.4-6.8] | 2.8 [1.41-5.618] |
| **CAH FSFI ≥ 26.55** | 0.63 [0.1-1.58] | 1.66 [0.4-3.06] | 4.7 [0.99-6.4] |
| **Controls FSFI < 26.55** | 1.13 [0.86-1.44] | 2.16 [1.99-4.175] | 1.2 [0.8-1.84] |
| **Controls FSFI ≥ 26.55** | 0.95 [0.9-1.195] | 1.75 [1.03-2.25] | 1.19 [0.84-1.31] |
| **All women with AED FSFI < 26.55** | 1.43 [0.7525-2.73] | 3.46 [1.9-4.44] | 2.2 [1.41-3.048] |
| **All women with AED FSFI ≥ 26.55** | 1.57 [1.16-2.125] | 3.06 [2.325-4.285] | 1.86 [1.41-2.57] |
| **All women FSFI < 26.55** | 1.33 [0.81-2.155] | 3.04 [1.905-4.405] | 1.95 [1.33-2.93] |
| **All women FSFI ≥ 26.55** | 1.36 [0.995-1.853] | 2.755 [2.243-3.685] | 1.77 [1.303-2.563] |

AED – androgen excess disorders, CAH – congenital adrenal hyperplasia, PCOS – polycystic ovary syndrome.
